# 161. Assessing Trends in 1,3-β-D Glucan and *Aspergillus* Galactomannan Antigen Ordering to Improve Use

**DOI:** 10.1093/ofid/ofac492.239

**Published:** 2022-12-15

**Authors:** Victoria Poplin, Rachael Liesman, Nathan C Bahr

**Affiliations:** University of Kansas Health System, Kansas City, Kansas; University of Kansas Health System, Kansas City, Kansas; University of Kansas Medical Center, Kansas City, Missouri

## Abstract

**Background:**

1,3-β-D Glucan (BDG) and *Aspergillus* galactomannan antigen (GM) are commonly ordered. Overutilization of these tests increases the incidence of false positive results, potentially leading to unnecessary follow up testing, procedures, and treatment. The aim of this quality improvement (QI) study was to improve our understanding of BDG and GM utilization at our institution.

**Methods:**

The test orders for BDG and GM were modified within our electronic medical record (EMR) to provide education and require selection of ordering reason from a list of criteria. Educational sessions were provided to non-transplant provider groups who order BDG and GM frequently. Total number of tests, test results, patient immunosuppressed status, ordering location, and provider specialty were compared for a 3-month period pre/post intervention. Ordering criteria was collected post-intervention. SPSS software was used for chi square tests comparing groups.

**Results:**

BDG and GM ordering practices were compared in the pre- and post-intervention timeframes (Table 1). There were no significant differences in the total number of tests, immune status of patients, ordering location or test results for BDG or GM. A significant decrease in hospitalist ordering was found (n=79 (pre), n=48 (post), p=0.006). Positive GM tests were rare in both periods (1.96%, 6/306 and 3.34%, 10/299). Ordering criteria for both tests were also evaluated in the post-intervention period (Table 2). The most common ordering criteria selected for BDG and GM were disseminated fungal infection 275/435 (63%) and invasive aspergillosis 241/299 (81%), respectively.

**Figure:**
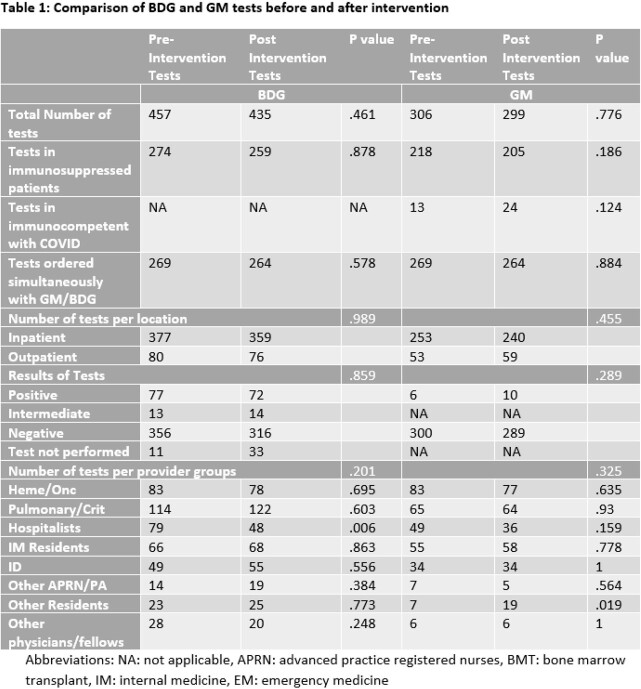

**Figure:**
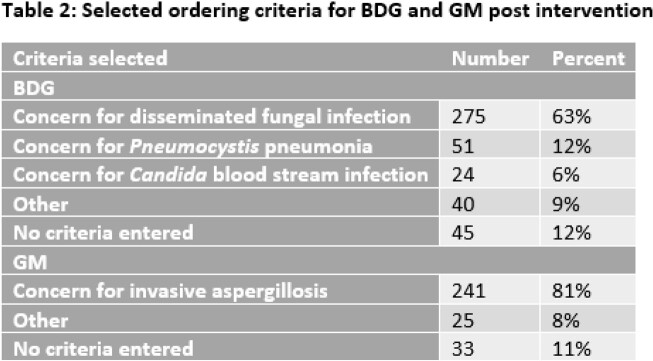

**Conclusion:**

Most BDG and GM tests were ordered utilizing appropriate ordering criteria and were among immunosuppressed patients. However, selection of an appropriate reason does not necessarily correlate to obtaining in the appropriate clinical setting. This QI project improved our understanding of how BDG and GM are utilized, and by whom. In the future, these results will be used to provide more targeted education to specific provider groups. In addition, further EMR order entry modifications will focus on potential false positive results to further optimize BDG and GM test utilization.

**Disclosures:**

**All Authors**: No reported disclosures.

